# Exploring the Role of Attachment Styles, Life Scripts, and Parental Mandates in Suicidal Behavior: Implications for Prevention and Intervention

**DOI:** 10.1192/j.eurpsy.2024.1621

**Published:** 2024-08-27

**Authors:** A. Romero Otalvaro, G. Galván Patrignani, E. P. Ruiz Gonzalez

**Affiliations:** ^1^Cordoba, Universidad de Cordoba, Monteria, Colombia; ^2^Anguilla, Global Humanistic University, curacao, Curaçao; ^3^Cordoba, Universidad Pontificia Bolivariana, Monteria, Colombia

## Abstract

**Introduction:**

The scientific literature widely acknowledges the multitude of factors contributing to suicide, emphasizing the intricate and dynamic interplay among genetic, biological, psychological, and social dimensions (Van Heeringen, 2001). Despite this consensus, each suicide case is unique, shaped by an exclusive combination of these factors. One relatively underexplored risk factor in the realm of suicidal behavior is attachment style. As posited by attachment theorists, avoidant and anxious/insecure attachment styles may hold predictive value for suicide attempts (Sheftall et al., 2014).

**Objectives:**

This study undertakes a comprehensive review of the relationships between attachment styles, life scripts, parental mandates, and suicidal behavior.

**Methods:**

This study delves into the interconnections between attachment styles, life scripts, parental mandates, and suicide, drawing from an extensive body of research and theory. A comprehensive review of existing literature was conducted to elucidate the intricate relationships among these variables and their potential influence on suicidal behavior.

**Results:**

The synthesis of existing research highlights a compelling link between attachment styles, life scripts, and parental mandates. Attachment styles, formed in early life, profoundly influence an individual’s interpersonal relationships, emotional regulation, and sense of self-worth. These attachment patterns lay the foundation for the development of life scripts—internalized narratives that dictate one’s beliefs, values, and expectations regarding their life course. Parental mandates, often transmitted explicitly or implicitly during childhood, further shape these life scripts by imposing conditions or constraints on the individual’s choices and aspirations.

Crucially, within this framework, suicidal behavior emerges as a possible outcome. Individuals with maladaptive attachment styles, burdened by parental mandates that discourage autonomous living or impose conditional acceptance, may perceive suicide as a way to escape perceived unmet expectations or alleviate emotional distress.

**Conclusions:**

This study underscores the intricate interplay between attachment styles, life scripts, parental mandates, and suicidal behavior. Understanding these complex relationships is pivotal in both prevention and intervention efforts. Recognizing the significance of family history, parental approaches, maladaptive beliefs, attachment patterns, and early caregiver interactions can inform the development of targeted strategies aimed at mitigating suicide risk in diverse contexts, including schools, communities, and clinical settings. By identifying these factors and their influence on suicidal behavior, practitioners and researchers alike can contribute to more effective prevention and intervention initiatives tailored to individual needs.

**Disclosure of Interest:**

None Declared

